# Supplementary material for: A Critical View of the Application of the APEX Software (Aqueous Photochemistry of Environmentally-Occurring Xenobiotics) to Predict Photoreaction Kinetics in Surface Freshwaters
Source: Molecules. 2019 Dec 18;25(1):9. doi: 10.3390/molecules25010009 (PMC7017383; doi:10.3390/molecules25010009)
Supplement: Supplementary file 1 [file molecules-25-00009-s001.zip › 3.2.4_gcc-4.4.0.zip!/3.2.4_gcc-4.4.0/3.2.4_gcc-4.4.0/doc/octave/html/interpreter/Mex_002dFiles.html]

Mex-Files - Untitled


Next: Standalone Programs,
Previous: Oct-Files,
Up: Dynamically Linked Functions


---

### A.2 Mex-Files

Octave includes an interface to allow legacy mex-files to be compiled
and used with Octave. This interface can also be used to share code
between Octave and non Octave users. However, as mex-files expose the
internal API of an alternative product to Octave, and the internal
structure of Octave is different to this product, a mex-file can never
have the same performance in Octave as the equivalent oct-file. In
particular to support the manner in which mex-files access the variables
passed to mex functions, there are a significant number of additional
copies of memory when calling or returning from a mex function. For
this reason, new code should be written using the oct-file interface
discussed above if possible.

- Getting Started with Mex-Files- Working with Matrices and Arrays in Mex-Files- Character Strings in Mex-Files- Cell Arrays with Mex-Files- Structures with Mex-Files- Sparse Matrices with Mex-Files- Calling Other Functions in Mex-Files
